# Supplementary material for: 3D virtual reconstruction of the Kebara 2 Neandertal thorax
Source: Nat Commun. 2018 Oct 30;9:4387. doi: 10.1038/s41467-018-06803-z (PMC6207772; doi:10.1038/s41467-018-06803-z)
Supplement: Supplementary file 1 — Supplementary Information [file 41467_2018_6803_MOESM1_ESM.pdf]

Supplementary Information for "3D virtual reconstruction of the Kebara 2 Neandertal thorax"

Gómez-Olivencia et al.

Table of contents

Preservation of the Kebara 2 thoracic elements 2

Supplementary Figures 1-5

Supplementary Note 1. Orientation of the transverse processes of the thoracic vertebrae 7

Supplementary Figures 6-7

Supplementary Tables 1-4

Multivariate analysis of the Kebara 2 thorax reconstructions 17

Supplementary Figures 8-9

Supplementary Note 2. Reconstruction of the Kebara 2 thorax 19

Supplementary Figures 10-20

Supplementary Table 5

Supplementary references 39

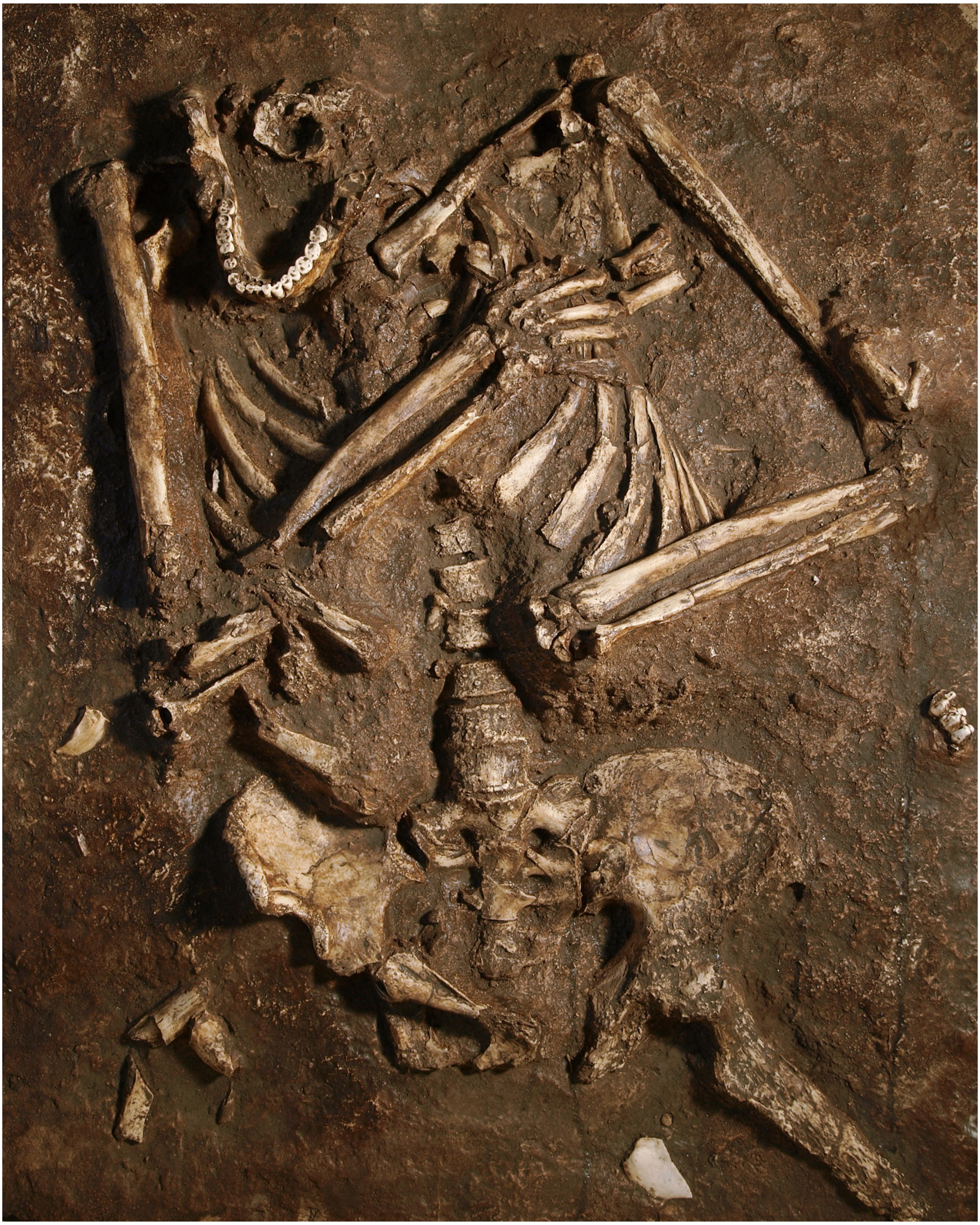

Supplementary Fig. 1. Photograph of the cast of the Kebara 2 skeleton as it was found which shows the preserved anatomical elements[1] (Photograph courtesy of J. Trueba/Madrid Scientific Films).

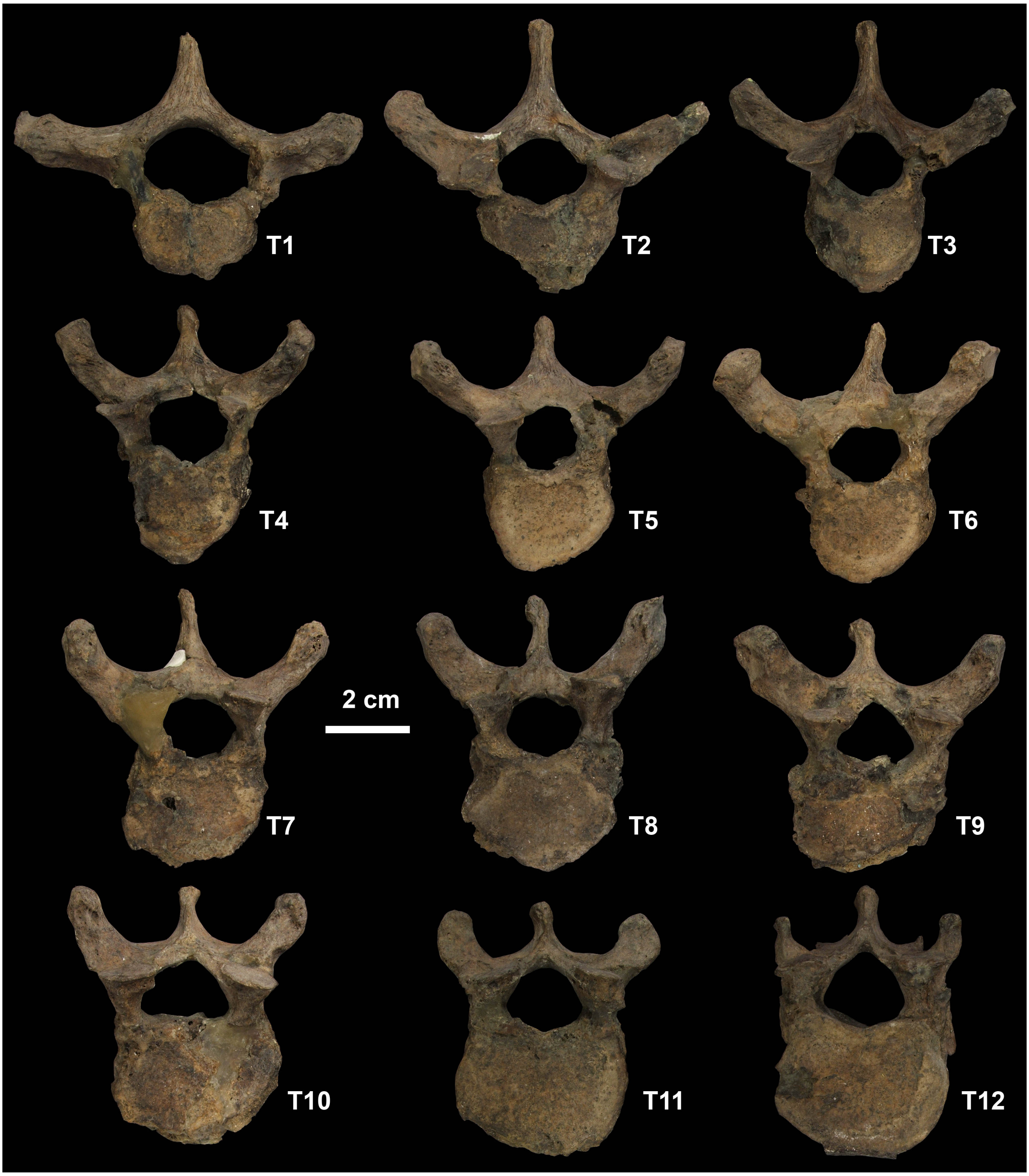

Supplementary Fig. 2. Cranial views of all the thoracic vertebrae of Kebara 2. Following the observations performed by Holliday (pers. comm.) we have swapped the position of the 7th and the 8th thoracic vertebrae (see below).

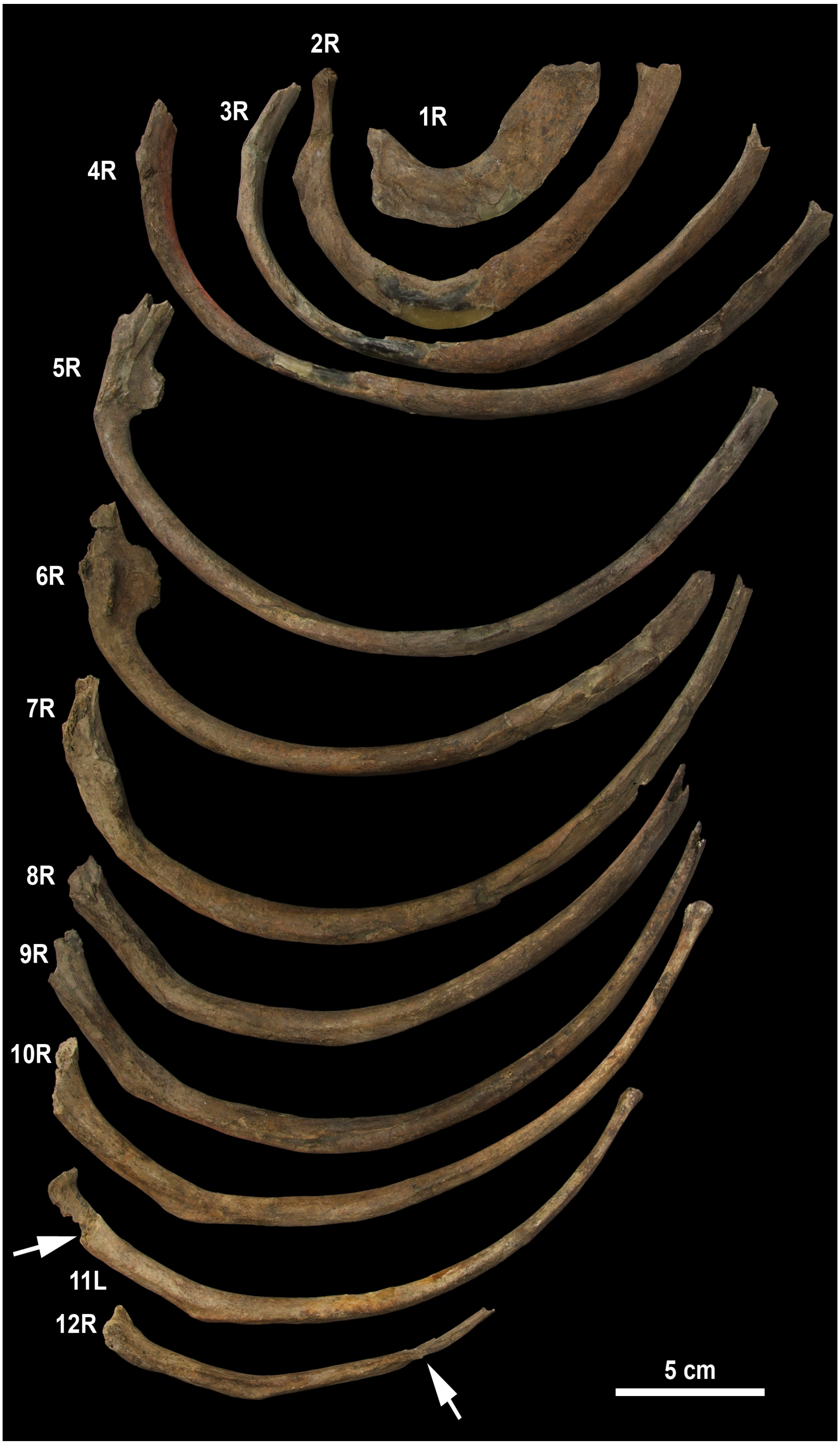

Supplementary Fig. 3. Cranial view of the ribs from the right side of Kebara 2. The arrows indicate errors in the reconstruction (see Supplementary Note 2).

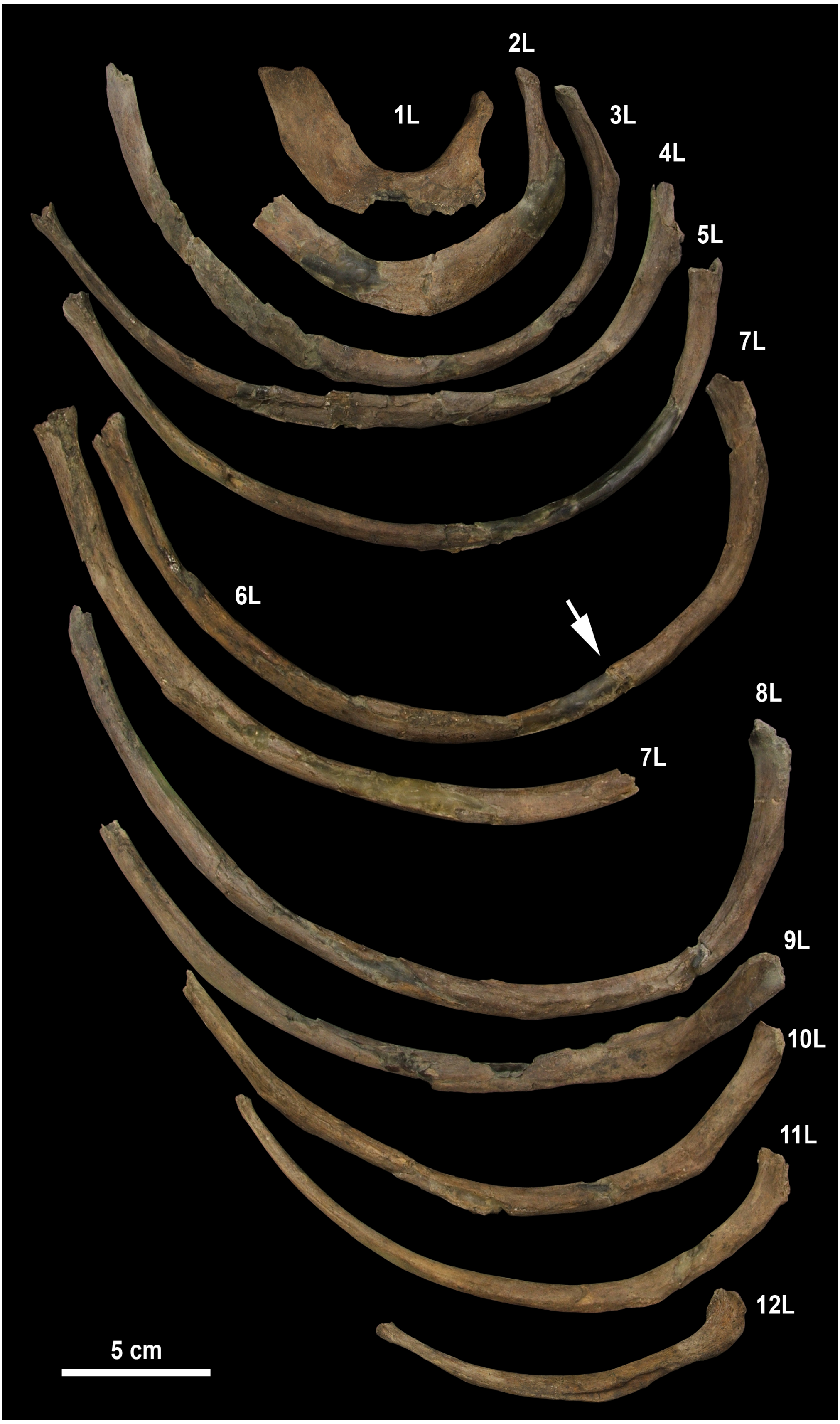

Supplementary Fig. 4. Cranial view of the ribs from the left side of Kebara 2. The arrow indicates an error in the reconstruction (see Supplementary Note 2).

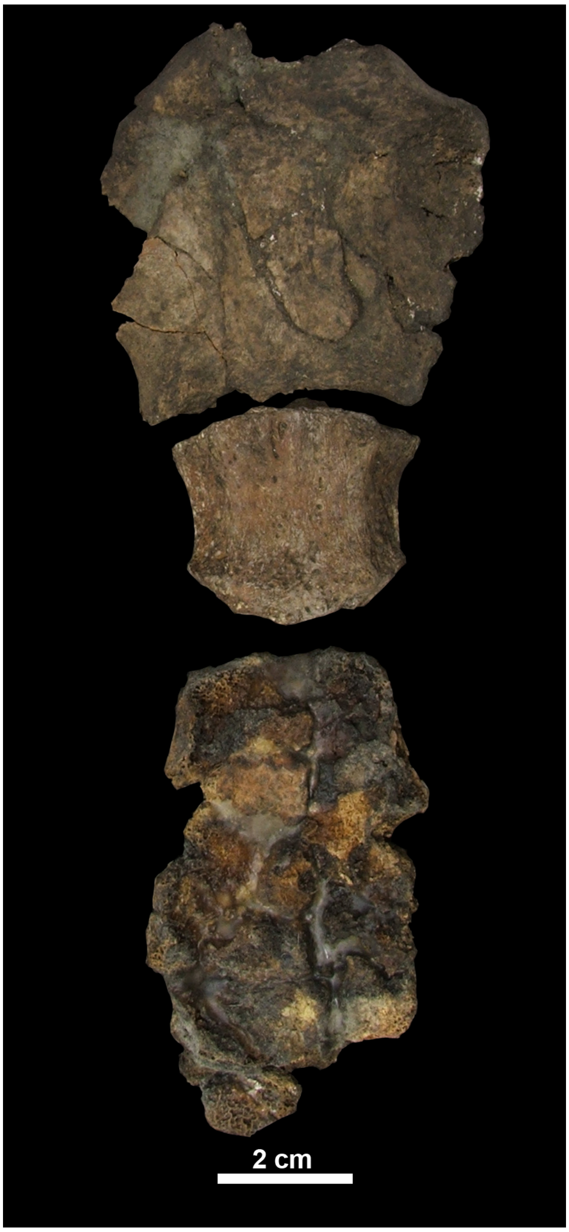

Supplementary Fig. 5. Ventral view of the sternum of Kebara 2.

Supplementary Note 1. Orientation of the transverse processes of the thoracic vertebrae

We have assessed the orientation of the transverse processes of the thoracic vertebrae T1-T10 (Supplementary Fig. 6). Starting in T4, Neandertals show, on average, more dorsally oriented transverse processes (SI Table 1). This morphology in Neandertals has been related to a more invaginated thoracic spine into the thorax[2], which we have confirmed in the 3D virtual reconstruction of this thorax. This may result in significantly smaller maximum transverse diameter of some thoracic vertebrae (Supplementary Table 2). Despite differences in orientation, in general, the length of the thoracic transverse processes of the thoracic vertebrae in Neandertals is similar to that of modern humans (Supplementary Table 3). Regarding Kebara 2, only in T8-T10 we have detected that the transverse process from the left side is longer (Supplementary Table 3), which results in more asymmetry than is present in our modern human sample (Supplementary Table 4). We have also noted that the transverse processes from the right side seem to be more robust, but have not been to quantify it (e.g., see Supplementary Fig. 7). These asymmetries could be related to the slight degree of scoliosis detected previously[3] and the pathology present in one of the sides of the costal skeleton[4,5].

In any case, the current Neandertal fossil record available to assess the orientation of the transverse processes is limited to a few individuals. Thus, more fossil specimens are necessary in order to fully understand the variation present in Neandertals.

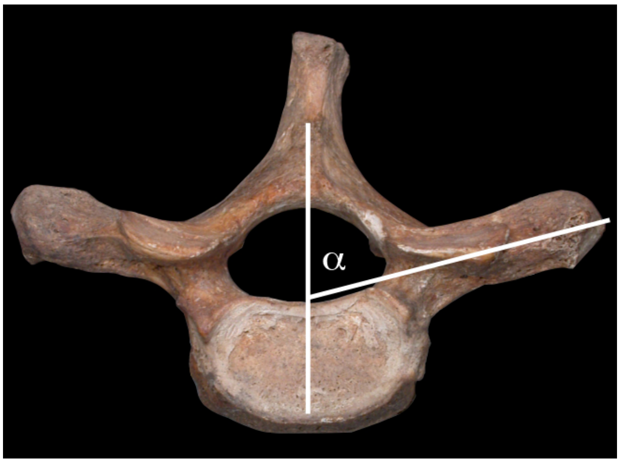

Supplementary Fig. 6. Measurement of the transverse process orientation in T1-T10. The orientation of the transverse process was measured relative to the mid-sagittal plane. Larger values of the angle α indicate more transversally oriented transverse processes while smaller values of the angle α indicate more dorsally oriented transverse processes.

Supplementary Table 1

Transverse process orientation[a] (in º) of the Kebara 2 thoracic vertebrae compared to other Neandertals and with our modern sample.[b]

Vertebra		Neandertals			Recent European males		
		Kebara 2	LC1	Regourdou 1	Right<br>Mean ± SD<br>(min-max)	Left<br>Mean ± SD<br>(min-max)	*n*
T1	right/left	78.3/74.6	-/72.1		78.0 ± 6.5 (67.9-92.5)	77.9 ± 5.6 (66.2-88.1)	28
	Total	152.9			155.9 ± 11.2 (138.1-174.9)		28
T2	right[c]/left	**<u>52.9*</u>**/(59.1)	(66.0)/(57.9)	58.3/58.9	64.0 ± 4.5 (54.1-72.9)	64.3 ± 5.1 (52.8-75.4)	30
	Total	112.0	(123.9)	117.2	128.3 ± 8.8 (107.6-147.9)		30
T3	right/left	58.3/54.2			58.1 ± 5.9 (44.9-70.3)	57.2 ± 5.4 (48.0-66.4)	32
	Total	112.5			115.3 ± 10.0 (93.7-135.3)		32
T4	right/left	**<u>44.5**</u>**/52.0			55.5 ± 4.0 (49.2-64.9)	55.8 ± 5.0 (48.3-66.5)	29
	Total	**<u>96.5</u>**			111.3 ± 8.1 (98.1-127.9)		29
T5	right/left	52.2/55.2			56.5 ± 6.0 (45.8-73.3)	57.2 ± 5.6 (46.2-66.5)	30
	Total	107.5			113.7 ± 10.5 (94.4-138.8)		30
T6	right/left	**<u>46.8*</u>**/51.4			57.7 ± 4.6 (47.4-66.6)	57.0 ± 5.7 (48.0-70.1)	30
	Total	98.2			114.7 ± 9.5 (97.2-136.6)		30
T7	right/left[c]	**<u>40.2**</u>**/50.7		**<u>52.2</u>**/-	58.8 ± 4.7 (52.9-69.8)	56.8 ± 5.4 (49.0-67.5)	27
	Total	**<u>90.9**</u>**			115.6 ± 8.8		27

					(102.0-134.6)		
T8	right/left[c]	**36.6**/42.8****	**44.9****/52.7		56.8 ± 3.6<br>(51.0-64.6)	57.0 ± 4.9<br>(47.6-66.4)	27
	Total	**79.4****	**97.6***		113.7 ± 8.0<br>(99.8-130.6)		27
T9	right/left	50.2/47.5	**(37.8**)**/47.6		56.6 ± 4.5<br>(50.0-68.3)	55.3 ± 6.4<br>(43.9-66.4)	29
	Total	97.7	**85.4****		111.9 ± 10.3<br>(94.3-134.5)		29
T10	right/left	**30.6****/41.8	40.4/44.3		50.2 ± 7.0<br>(39.8-65.9)	50.2 ± 6.7<br>(37.5-62.5)	26
	Total	**72.4***	84.7		100.4 ± 12.6<br>(77.3-123.2)		26

LC1 = La Chapelle-aux-Saints 1

[a]The right/left values indicate the orientation from the mid-sagittal plane in cranial view, while the total value indicates the orientation of the transverse process to one another. Larger values (as in T1) indicate more laterally oriented transverse processes, while smaller values indicate more dorsally oriented transverse processes (Supplementary Fig. 6).

[b]Including the results of the z-score test between each Neandertal individual and our modern human male sample

For the z-score test, the values indicated by an asterisk are significantly different from the modern male comparative sample (* = $p < 0.05$; ** = $p < 0.01$). Values underlined are outside the range of the modern human comparative sample. Values in parentheses are estimated.

[c]The orientation of the left transverse process may be affected by the dorsal displacement (due to taphonomic processes) of this region. However, the orientation of the antimeres is still below the modern human sample.

Supplementary Table 2

Maximum transverse diameter[a] (in mm) of the Kebara 2 thoracic vertebrae compared to other Neandertals and to a modern human sample.[b]

Thoracic vertebra	Neandertals			Recent male sample[c]	
	Kebara 2	LC1	Regourdou 1	Mean ± SD (min-max)	*n*
T1	79.7			75.8 ± 3.5 (69.6-83.5)	59
T2	71.9		70.6	71.1 ± 3.3 (62.7-79.5)	62
T3	66.8			65.5 ± 3.6 (53.4-73.5)	66
T4	**55.8****			63.6 ± 3.0 (55.8-69.7)	62
T5	58.9			64.1 ± 3.2 (56.8-73.1)	63
T6	62.0			65.0 ± 3.2 (57.9-72.4)	63
T7	**58.9***			65.5 ± 3.0 (58.9-72.9)	59
T8	**57.3***	62.5		63.8 ± 3.1 (56.6-71.1)	60
T9	62.2	62.3		62.2 ± 3.5 (54.4-69.8)	62
T10	55.1	57.0		58.7 ± 4.2 (50.3-69.4)	59

LC1 = La Chapelle-aux-Saints 1

[a]Maximum transverse diameter: maximum transverse diameter between the most lateral points of the transverse processes[6].

[b]A z-score analysis has been performed between each Neandertal individual and our modern human male sample. Values in bold and indicated with an asterisk are significantly different from the modern male comparative sample (* = $p < 0.05$; ** = $p < 0.01$).

[c]The recent male sample comprises a total of 41 Europeans (University of Burgos, Burgos, Spain) and 26 Euroamericans (Hamman-Todd collection, Cleveland Museum of Natural History, Cleveland, USA).

Supplementary Table 3

Maximum length of the transverse process[a] (in mm) of the Kebara 2 thoracic vertebrae compared to other Neandertals and with our modern male sample.[b]

Vertebra	Side	Neandertals				Recent male sample	
						Right transverse process	
		Kebara 2	LC1	La Ferrassie 1	Regourdou 1	Mean ± SD (min-max)	*n*
T1	right/left	29.8/-	-/(30.0)	-/29.9		28.7 ± 2.0 (25.0-38.1)	63
T2	right/left	29.2/30.0	/(31.0)	27.3/-	29.4/29.4	29.4 ± 1.4 (25.7-31.8)	63
T3	right/left	27.8/28.4	(30.5)/-			28.2 ± 1.5 (24.9-31.4)	65
T4	right/left	27.0/25.7				27.9 ± 1.3 (25.3-32.2)	56
T5	right/left	27.3/28.3				28.3 ± 1.4 (24.8-31.9)	64
T6	right/left	30.6/29.8				28.9 ± 1.5 (26.2-32.1)	64
T7	right/left	-/29.3			**26.6***/-	29.2 ± 1.3 (27.1-31.9)	61
T8	right/left	28.9/**31.5***	(28.7)/29.3			28.2 ± 1.6 (24.7-31.9)	60
T9	right/left	28.5/**31.7***	-/26.9			27.8 ± 1.6 (24.1-32.2)	61
T10	right/left	28.5/**30.2***	26.0/-			26.2 ± 1.7 (22.4-31.4)	61

LC1 = La Chapelle-aux-Saints 1

[a]Measured from the internal part of the pedicle to the most distal tip of the transverse processes.

[b]Including the results of the z-score test between each Neandertal individual and our modern human male sample

For the z-score test, the values with an asterisk are significantly different from the modern male comparative sample (* = $p < 0.05$; ** = $p < 0.01$). Values underlined are outside the range of the modern human comparative sample. Values in parentheses are estimated.

Supplementary Table 4

Percentage asymmetry[a] between the maximum length of the transverse process.

Vertebra	Kebara 2	Recent Europeans
		**Mean ± SD (Min-Max) *n***
T8	9.0	4.0 ± 2.7 (0.3-9.6) *n* = 33
T9	**11.2****	3.0 ± 3.0 (0.0-13.7) *n* = 31
T10	6.0	3.6 ± 2.8 (0.0-10.7) *n* = 30

[a]Percentage asymmetry was calculated as the absolute difference in length between right and left sides, divided by the smaller side, and multiplied × 100[7,8].

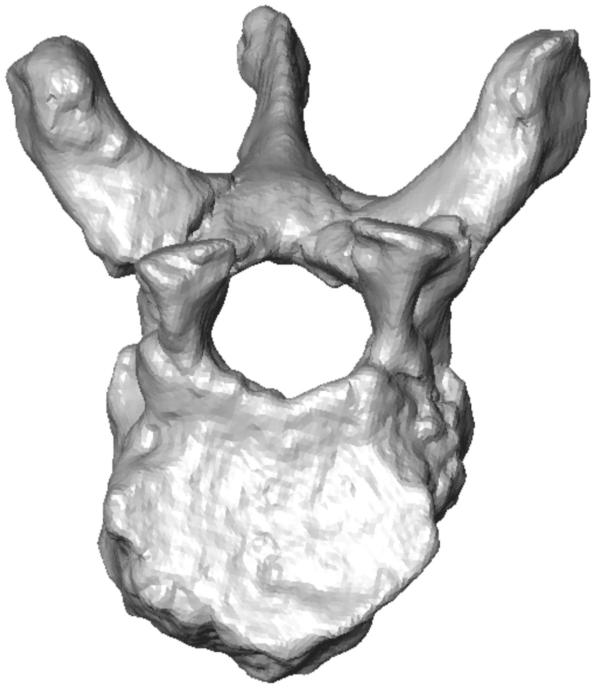

Supplementary Fig. 7. Cranial view of Kebara 2's T8 (T7 according to Arensburg[9]). Note the more robust right transverse process compared to the left side one.

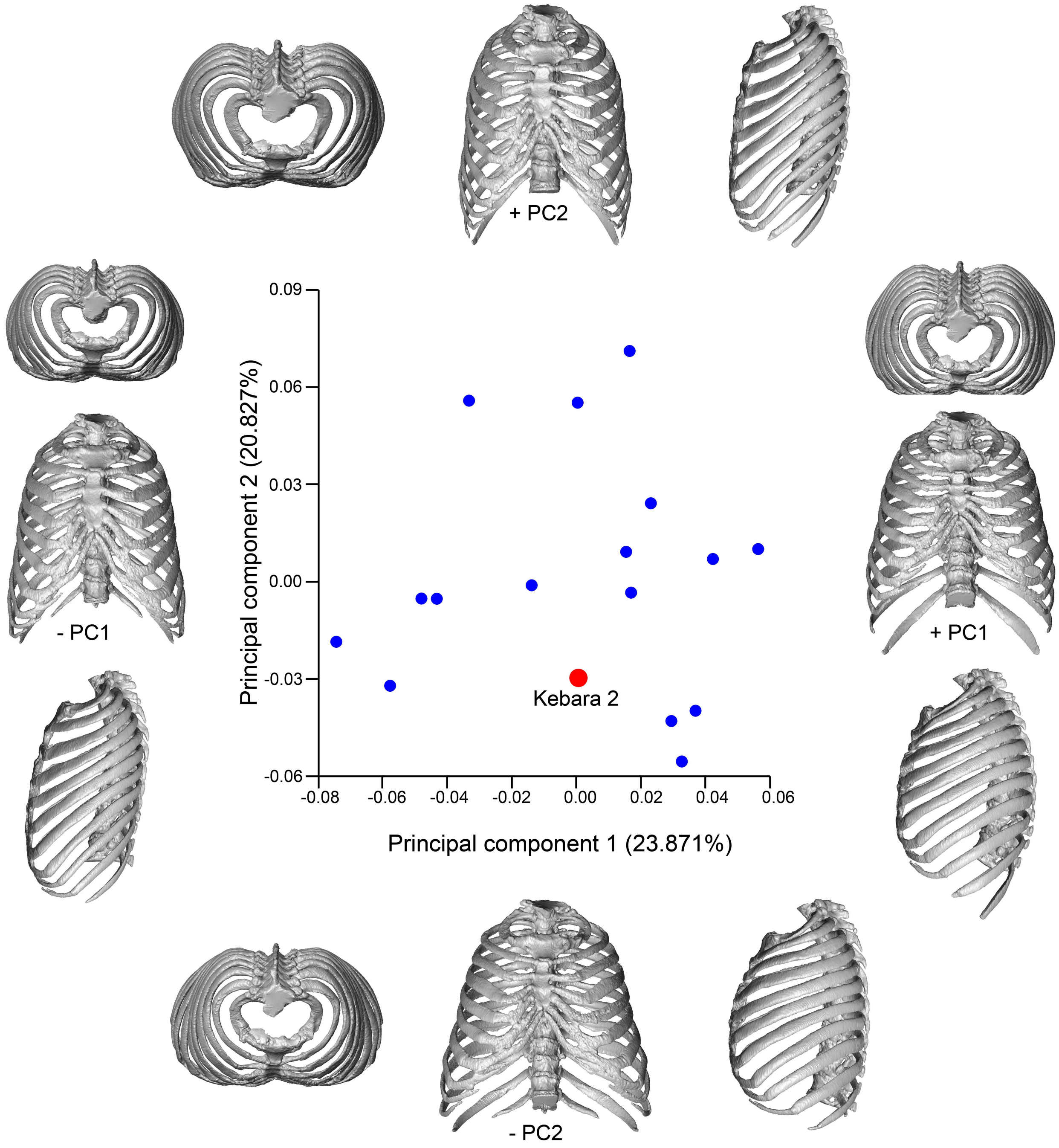

Supplementary Fig. 8. Scatterplot of principal component scores (PC1 and PC2) in shape space. In this scatterplot Kebara 2 is within the modern male sample range of variation, but at the lower limits of the male range of variation for PC2.

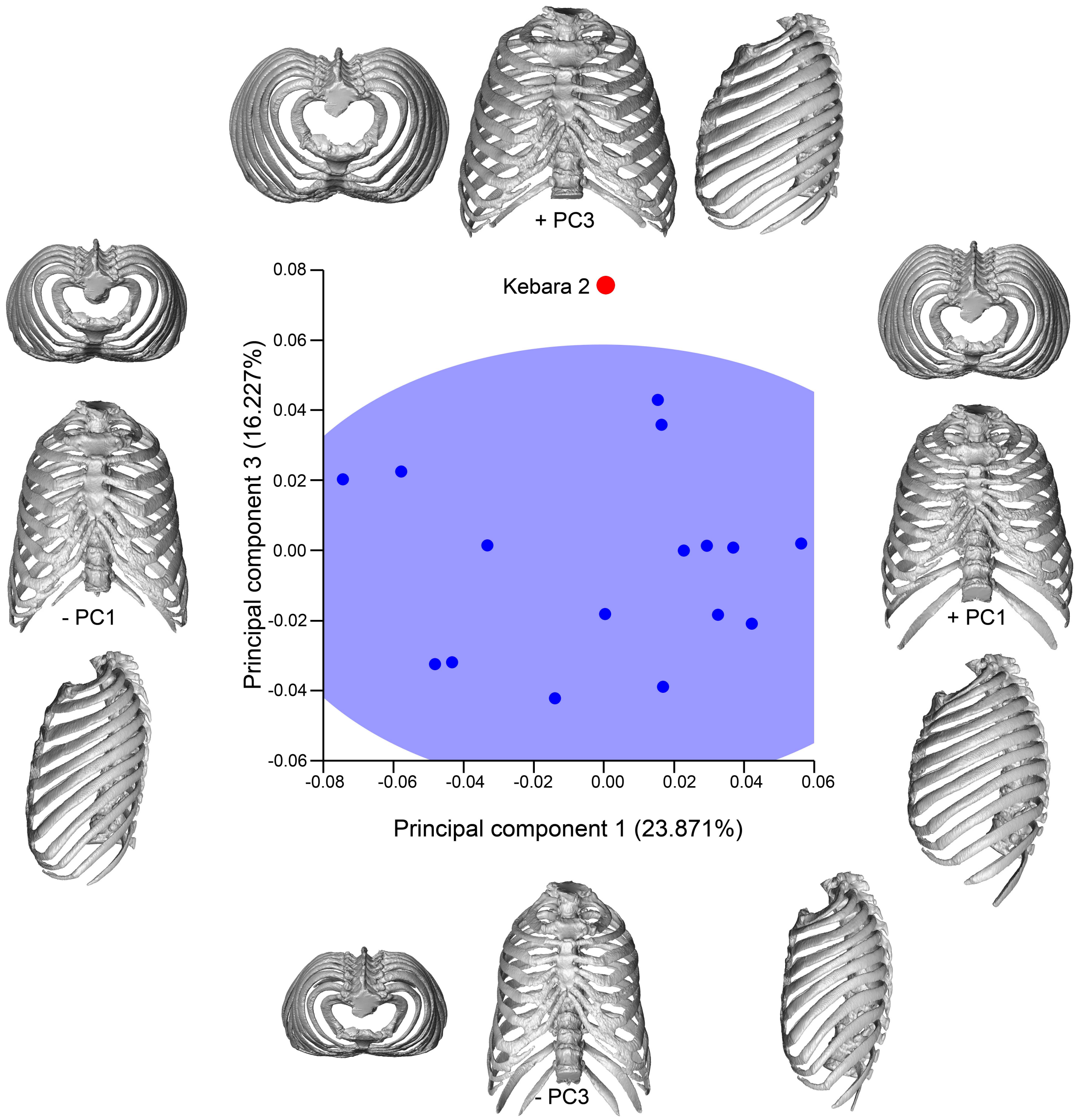

Supplementary Fig. 9. Scatterplot of principal component scores (PC1 and PC3) in shape space. In this scatterplot Kebara 2 shows very positive values of PC3, outside the 95% equiprobability ellipse of the male variation.

Supplementary Note 2. Reconstruction of the Kebara 2 thorax

The virtual reconstruction of the Kebara 2 (K2) thorax is built upon the reconstruction of the vertebral column of this individual[2], although we have slightly modified this spine reconstruction. Then, to this reconstruction we have added the individual ribs, the sternum, and the coxal bones.

The vertebral column

We have slightly modified a previous reconstruction of the spine of Kebara 2 Neandertal[3] in the following ways:

<u>Summary of the reconstruction of the vertebral column[2]</u>

-The spatial orientation of the sacrum was reconstructed using two approaches: a) the pelvic incidence, which measures the orientation of the sacral endplate in relation to the acetabulum[10,11]; and b) the sacral anatomical orientation (angle γ[10]). The mean value of these two methods yielded a value of 21º to the horizontal for K2, significantly below the c. 40º present in modern humans[3]. Thus, the K2 sacrum shows a more horizontally oriented sacral endplate, which is also related to a more vertical sacrum.

-Then, the lumbar and thoracic vertebrae were put into place making sure that they were properly articulated with one another. These vertebrae were aligned following the estimation of the lordotic and kyphotic curvatures in the mid-sagittal plane expected on this individual based on the vertebral morphology.

-The lumbar vertebrae were aligned using the mean of the two published lumbar lordosis estimations (27º). The first value (25º) was estimated based on the relationship between lordosis and the orientation of the inferior articular processes of the lumbar vertebrae to the vertebral body [12]. The second one (29º) was based on the relationship between the pelvic incidence and lumbar lordosis[11,12]. These values are below the mean values of modern humans, although still within known species variation. Thus, K2 shows less lordosis than do modern humans.

-The thoracic vertebrae were aligned using the value of the kyphotic angle using the method developed by Goh et al.[13]. This method is based on the wedging of the vertebral bodies of the thoracic vertebrae, measured as a ratio between the ventral and dorsal cranio-caudal diameters (heights) of the thoracic vertebral bodies. K2 yielded a kyphotic angle of 44º which is slightly below, but similar to, modern human samples[3]. Additionally, a mild asymmetry of the thoracic spine of K2 in the coronal plane was detected. Thus, K2 shows a kyphotic angle similar to that of modern humans and a mild scoliotic curve.

Differences from Been et al.'s[3] reconstruction

-For all the presacral vertebrae, we had access to better quality 3D reconstructions. These new reconstructed vertebrae were placed following Been et al.'s[2] reconstruction.

-Following the observations performed by Holliday (pers. comm.) we have swapped the position of the 7th and the 8th thoracic vertebrae (Supplementary Fig. 2).

-The spinous process was broken in the eighth thoracic vertebra (T8; Arensburg's T7) and inaccurately reconstructed. We have re-oriented this spinous process so it is resembles those of the vertebrae above and below it (Supplementary Fig. 10).

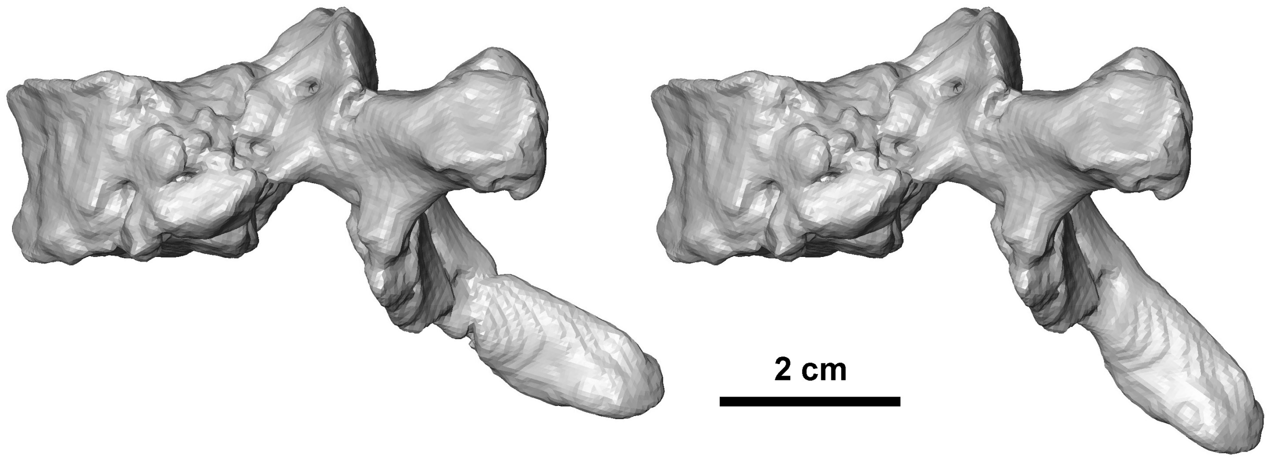

Supplementary Fig. 10. Left lateral view of the eighth thoracic vertebra (T8; T7 according to Arensburg[9]) before (left) and after (right) correction to the orientation of the spinous process.

-The right transverse process of the fourth lumbar vertebra (L4) was reconstructed by mirror-imaging its left-side counterpart (Supplementary Fig. 11).

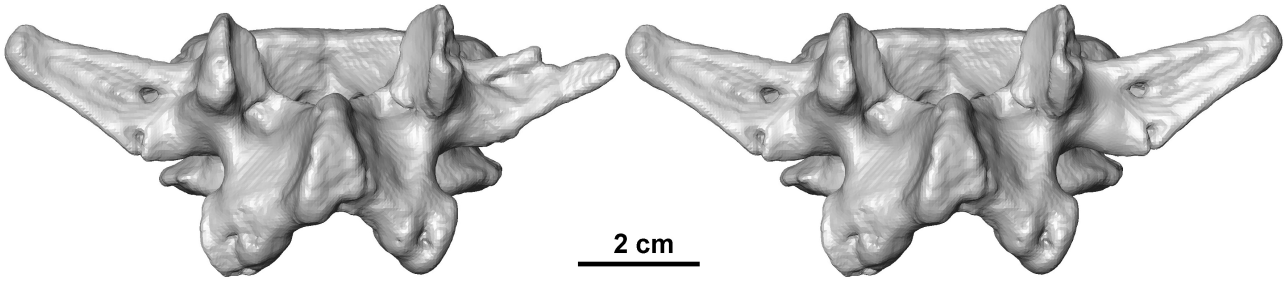

Supplementary Fig. 11. Dorsal view of the fourth lumbar vertebra (L4) before (left) and after (right) reconstruction of the right transverse process through the mirror-image of its left side counterpart.

The pelvis

-The pelvis was reconstructed in order to understand the thorax morphology within the context of the morphology of the whole trunk. The right innominate, which is the most complete one, was used for both right and left sides after mirror-imaging of it. The bi-iliac breadth obtained from this model (319.7 mm) is 6.7 mm larger than the previously published value of 313 mm[14].

The costal skeleton

-Previous work on the costal skeleton of K2 has noted the presence of errors in the reconstruction of the ribs as well as some degree of taphonomic distortion on the K2 skeleton, which affects both the spine and the costal skeleton[15]. Subsequent study of the original skeleton and the present virtual reconstruction has allowed us to detect new errors in the reconstruction in two ribs as well as to understand better the degree of taphonomic distortion of the K2 costal skeleton. Note that by "original" here and in later sections we mean the original reconstruction of the costal fragments.

-The K2 burial was found at 7.8 m of depth. The weight of the sediment covering the skeleton resulted in some damage to the costal skeleton. Most of the ribs are broken into different fragments. In some cases, the fragments were easily reconstructed following the original costal morphology. In other cases, the physical reconstruction did not properly follow the original anatomy, but a realignment was possible.

-In some cases, the rib shaft is, however, collapsed and broken into multiple pieces, which makes it impossible to correct the displacement between the fragments. In these cases, we have used the better-preserved antimere and we have mirror-imaged it.

-A previous study[15] detected an error in the reconstruction that affected ribs 6 and 7 on the left side. Here we have detected two additional reconstruction errors in K2 skeleton: the 11th and the 12th ribs on the right side (see below for details).

Ribs used in the 3D reconstruction of the K2 thorax

A summary of the ribs used in the 3D reconstruction of the K2 thorax and the degree (if any) of reconstruction performed can be found in Table S5.

Supplementary Table 5

Summary of the ribs used and the reconstruction performed on these ribs in order to complete the 3D virtual reconstruction of the Kebara 2 thorax. "Original" means the original reconstruction of the costal fragments; "reconstructed" indicates changes documented in previous works [15] or made in this study.

Left side	Rib	Right side
Reconstructed: 1R's shaft and 1L's head and neck (mirror imaged)	**1**	Reconstructed: 1R's shaft and 1L's head and neck
Mirror-imaged of 2R	**2**	Original
Mirror of the reconstructed 3R	**3**	Reconstructed
Mirror image of the reconstructed 4R	**4**	Reconstructed
Reconstructed	**5**	Reconstructed
Original	**6**	Reconstructed
Reconstructed	**7**	Original
Reconstructed	**8**	Original
Mirror image of 9R	**9**	Original
Mirror image of the reconstructed 10R	**10**	Original, only slightly reconstructed: a bone chip that was missing in the posterior angle was added virtually
Original	**11**	Mirror image of 11L
Original	**12**	Mirror image of 12L

First ribs

The first right rib (1R) was completed using the mirror image of the head and neck of its left-side counterpart (Supplementary Fig. 12). The shaft of 1R was selected for two reasons: first, it is the most complete side; and second, it follows better the Neandertal anatomy of antero-posteriorly straight ribs[16,17,18]. This completed 1R was then mirror-imaged in order to obtain 1L.

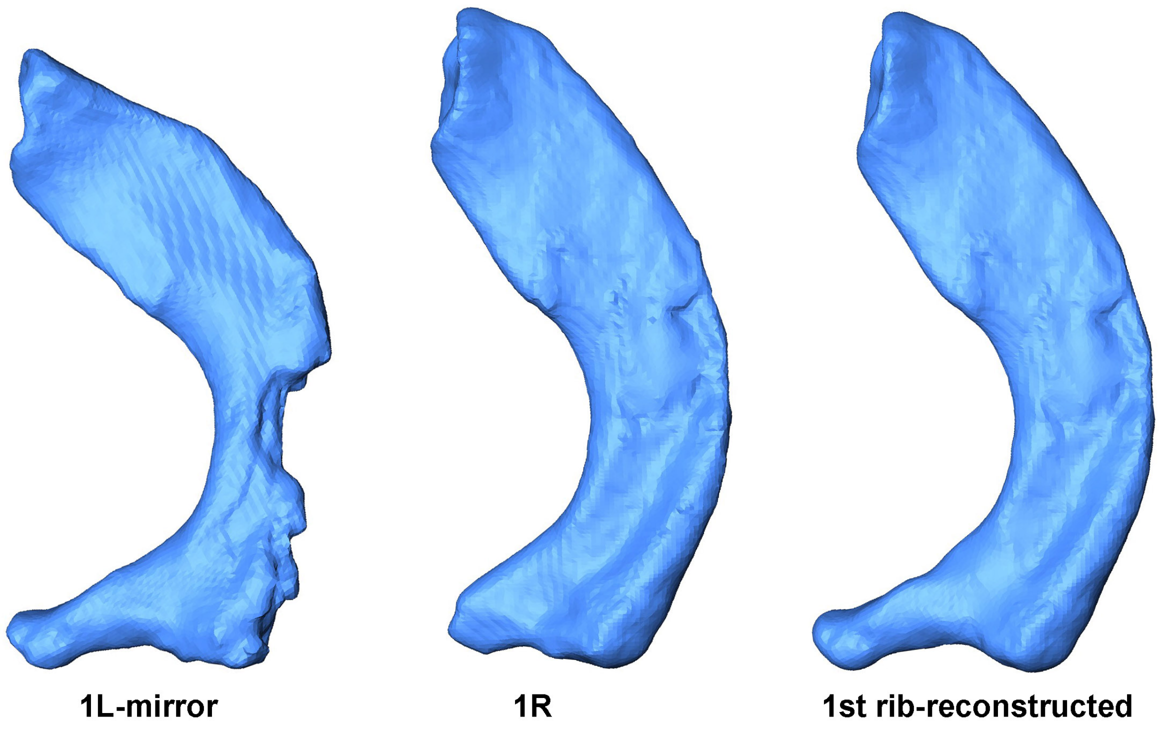

Supplementary Fig. 12. Process of reconstruction of the first rib. The shaft of the right rib was chosen because it was more complete and also less curved than its left side counterpart.

Second ribs

The original second rib from the right side (2R) was used in the 3D thorax reconstruction. Due to the incompleteness of the second rib from the left side, in the 3D thorax reconstruction we mirrored 2R.

Third ribs

In order to complete the missing parts of the vertebral end of the right third rib (3R), the head, neck, tubercle and the part of the shaft adjacent to the posterior angle from the third rib of the left side (3L) have been mirror imaged (Supplementary Fig. 13).

In the 3D thorax reconstruction, the 3L is the mirror image of the reconstructed 3R. The sternal half of the shaft was slightly moved ventrally to keep the anatomical congruence with the rest of the ribs from the left side.

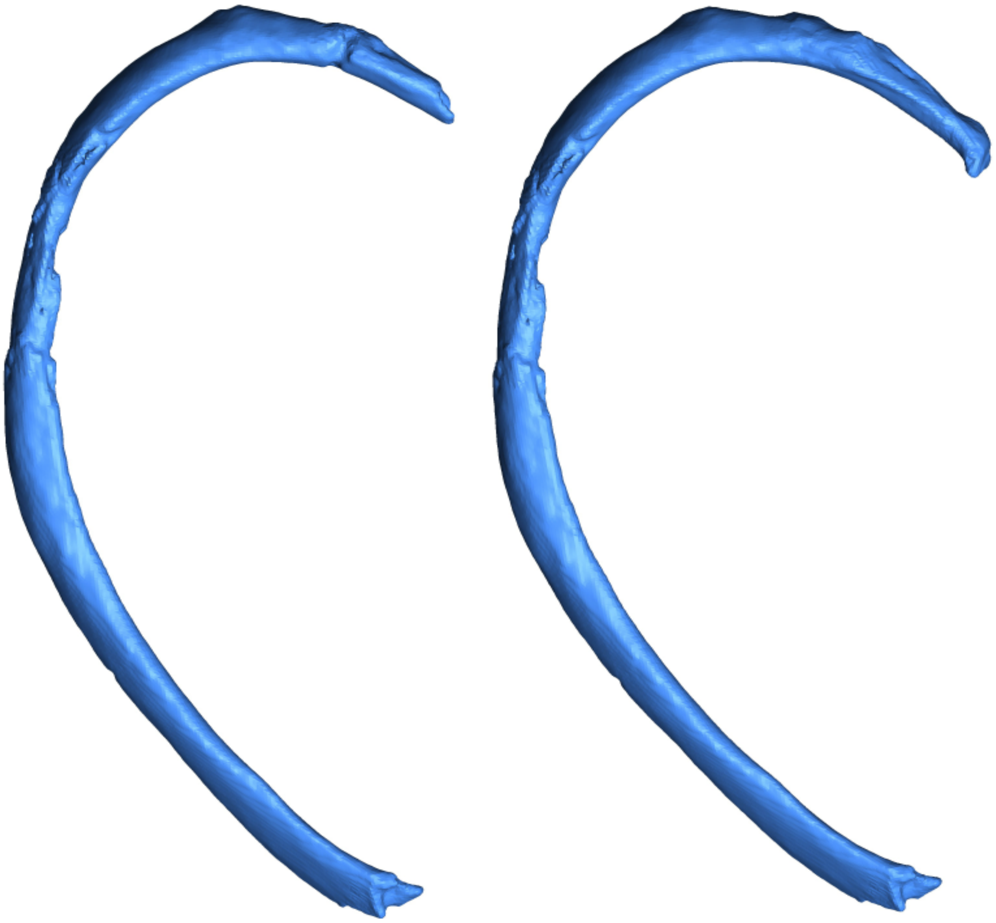

Supplementary Fig. 13. Original third rib from the right side (3R) of K2 (left) and reconstructed 3R after adding the mirror-imaged rib fragment from 3L (comprising head, neck, tubercle and the part of the shaft adjacent to the posterior angle) (right).

Fourth ribs

The original fourth rib from the right side (4R) was used in the 3D thorax reconstruction; however, the two main fragments that compose the fourth right rib have been realigned. The original specimen shows a very low curvature because the two main fragments that form this rib were not correctly realigned. When using the original rib in our 3D model, once the articular tubercle was put into place the sternal half of the rib did not follow the proper anatomical alignment of the ribs. We corrected this by rotating at the fracture line the sternal half of the rib inwards (posteriorly or internally) (Supplementary Fig. 14).

For 4L, the realigned 4R was mirror-imaged. However, in order to further improve the anatomical congruence with the adjacent ribs, the sternal end of the shaft was rotated outwards (anteriorly or externally).

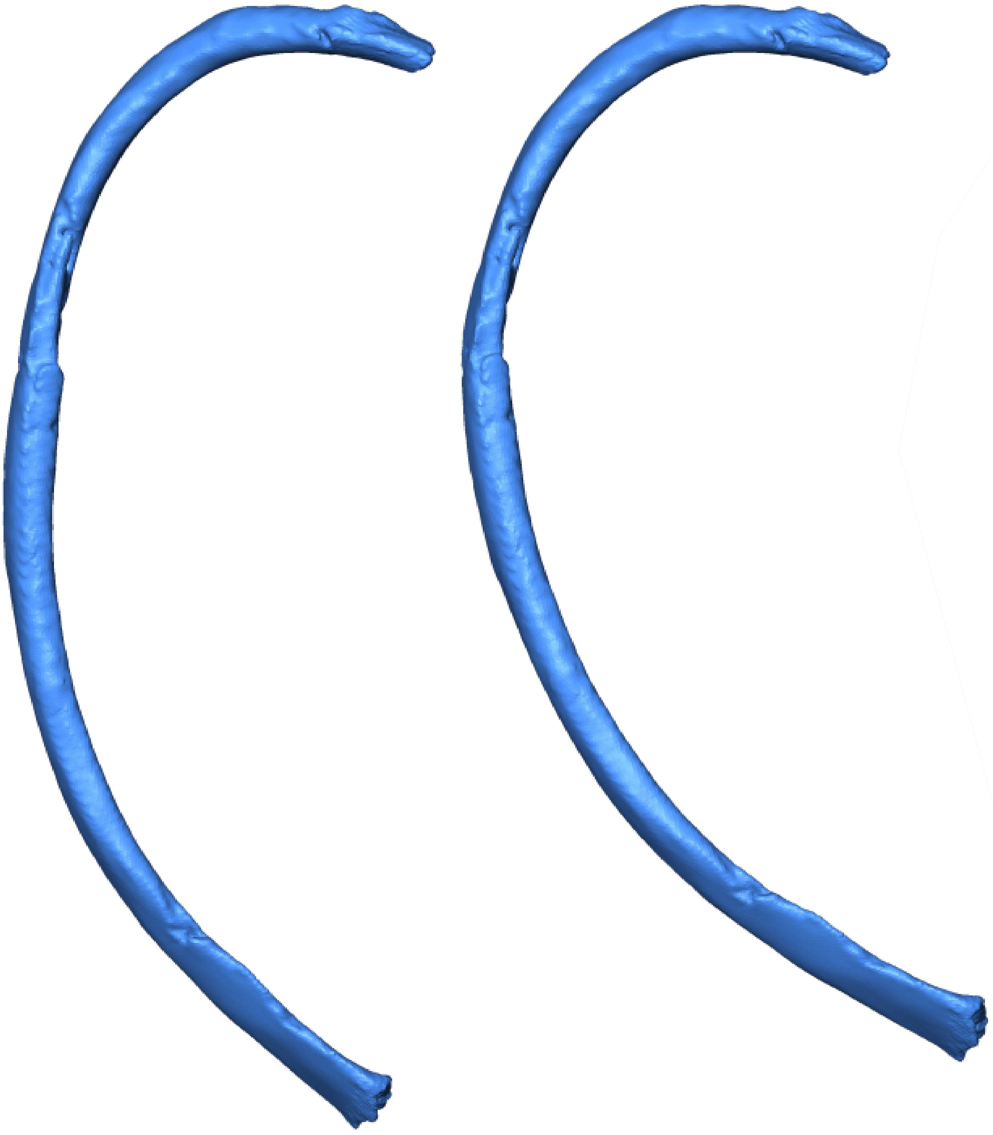

Supplementary Fig. 14. Original fourth rib from the right side (4R) of K2 (left) and 4R after realigning (rotating inwards) the sternal half of the shaft giving it more curvature.

Fifth ribs

The original fifth rib from the right side (5R) was used in the 3D thorax reconstruction; however, the sternal-most fragment was slightly rotated outwards to better fit with the morphology of adjacent ribs (Supplementary Fig. 15).

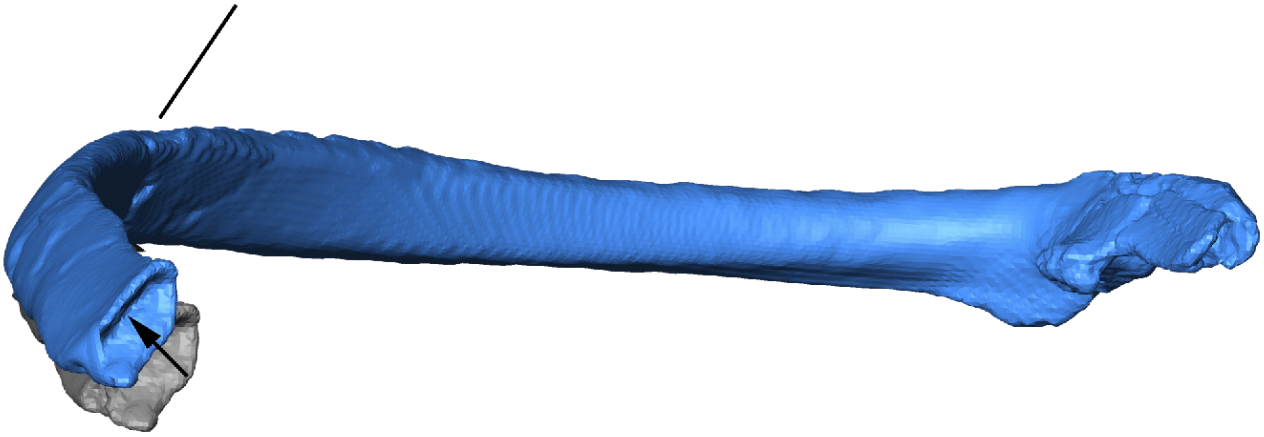

Supplementary Fig. 15. Original fifth rib from the right side (5R) of K2 before (grey) and after (blue) realigning the sternal-most fragment. The arrow marks the orientation of the realignment and the black line marks approximately the axis of the rotation.

The original fifth rib from the left side (5L) was used in the 3D thorax reconstruction. However, the sternal-most half of the rib was rotated outwards to better fit with the morphology of adjacent ribs (Supplementary Fig. 16).

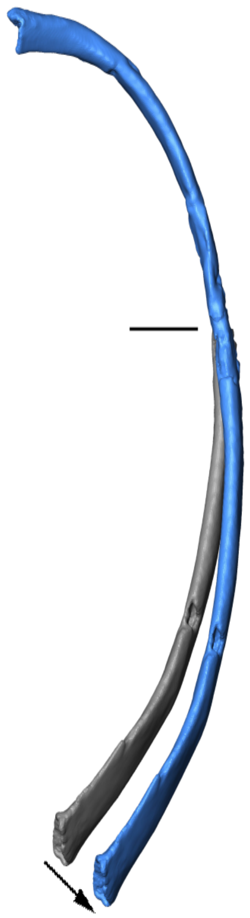

Supplementary Fig. 16. Original fifth rib from the left side (5L) of K2 before (grey) and after (blue) realigning the sternal-most fragment. The arrow marks the orientation of the realignment and the black line marks approximately the point of the rotation.

Sixth, seventh and eighth ribs from the right side (6R, 7R and 8R)

The original sixth, seventh and eighth ribs from the right side (6R, 7R and 8R) were used in the 3D thorax reconstruction.

g) Sixth and seventh ribs from the left side (6L and 7L)

The 6L from the K2 original collection (i.e., that published by Arensburg[8]ased on the shaft morphology at the refitting point and based on the comparisons with the proximal part of 7R, we concluded that the vertebral third of 6L (preserving the head, neck, tubercle, posterior angle and a portion c. 25mm of the shaft) is actually a right seventh rib[15,19]. The vertebral third of the 6L of the original collection better refits with the 7L of the original collection[15,19] (Supplementary Fig. 17).

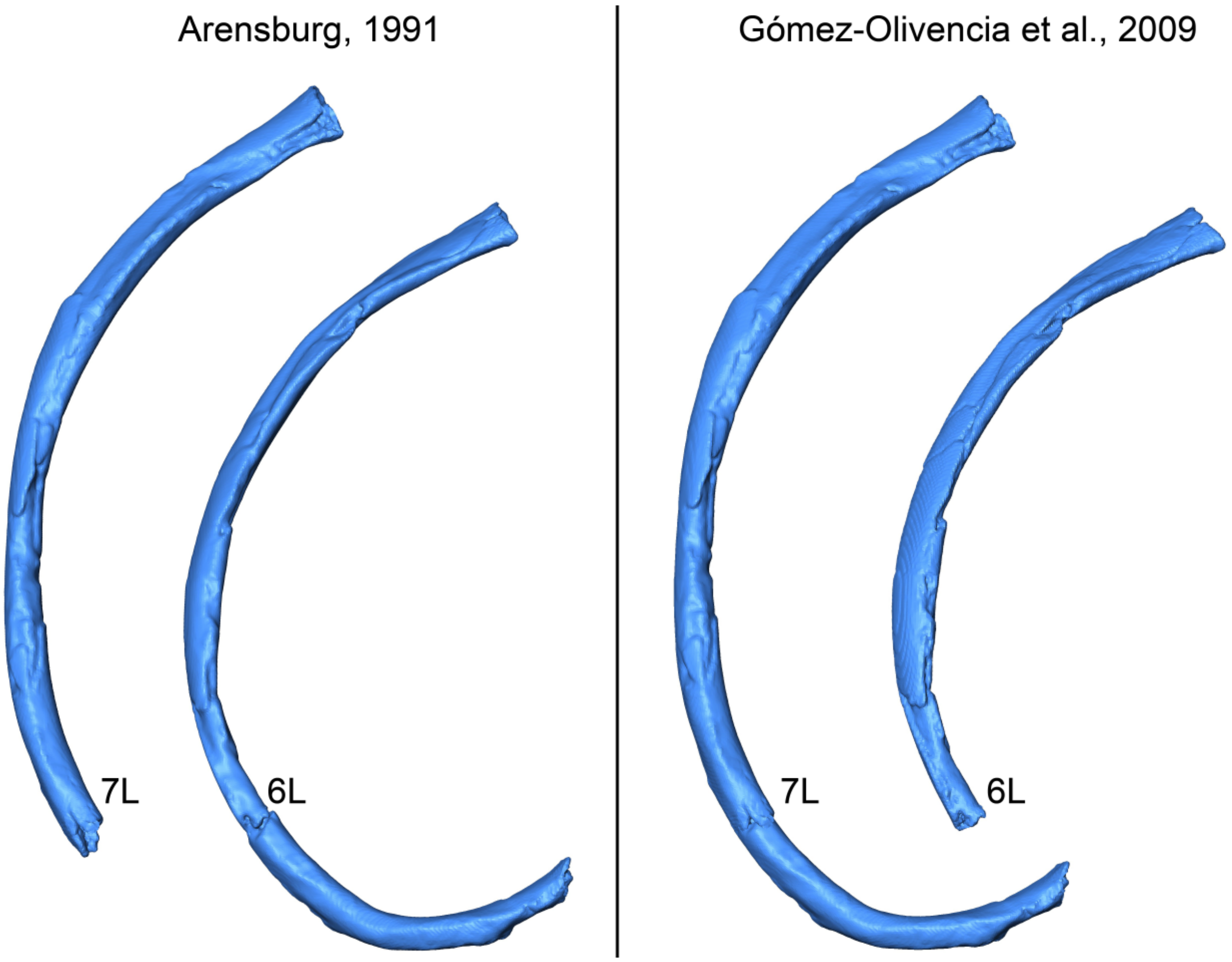

Supplementary Fig. 17. Cranial view of the sixth and seventh ribs from the left side following Arensburg[8] and Gómez-Olivencia et al.[15].

We have also observed that the left transverse process of the 7th thoracic vertebra (T7) is taphonomically distorted and located too dorsally. Thus, we have slightly displaced the seventh left rib (7L) to compensate for this.

Eighth rib from the left side (8L)

The original eighth rib from the left side (8L) was not used in the 3D thorax reconstruction because this specimen shows reconstruction and taphonomic problems that we could not resolve. It was not possible to properly realign the three main fragments that compose this fossil. Thus, we used the mirror-image of 8R to which the sternal-most part of the preserved diaphysis of the original 8L was added in order to complete this reconstruction (Supplementary Fig. 18).

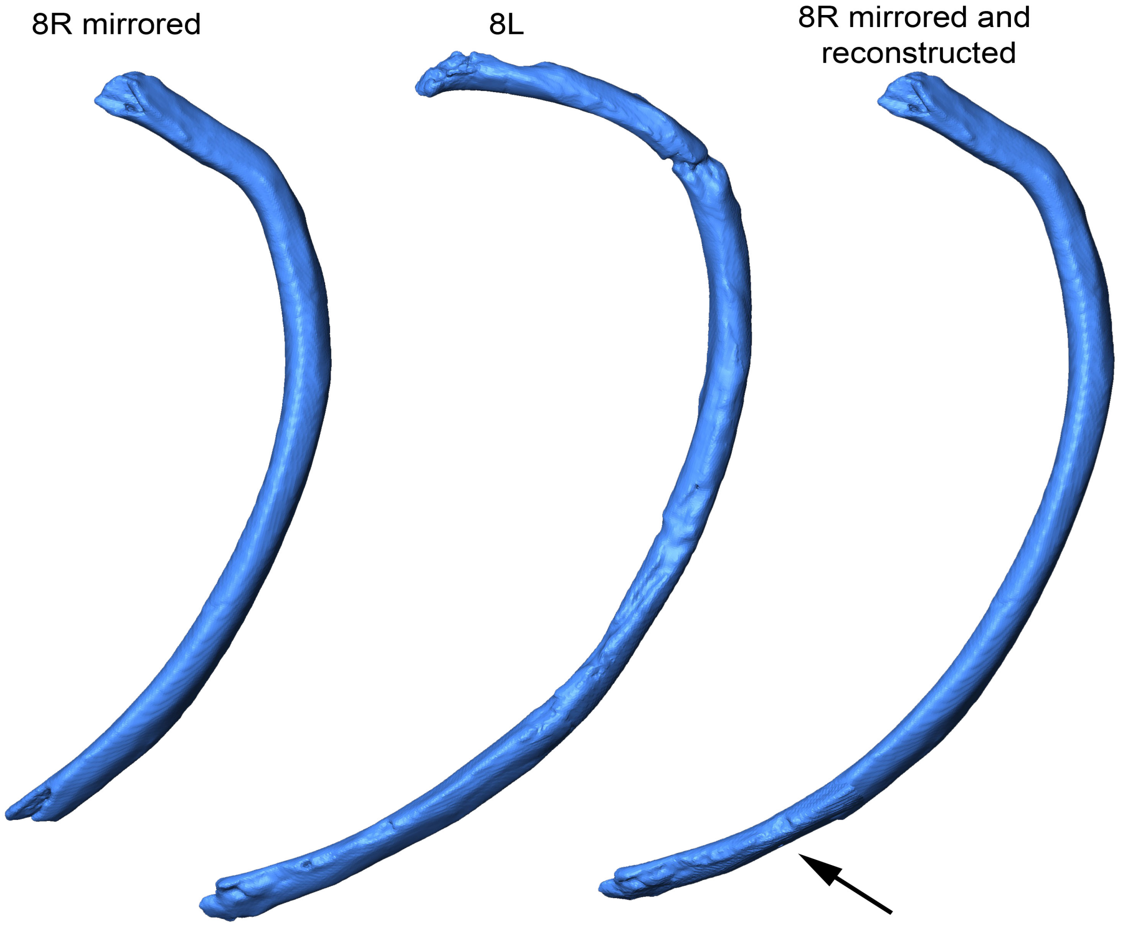

Supplementary Fig. 18. Due to taphonomic and reconstruction problems, the original eight rib from the left side (8L; center of the image) was not used. Instead the mirror image of the eight rib from the right side (left of the image) was used, to which a sternal fragment of the rib shaft was added to complete it (arrow).

Finally, and similarly to what happens in the suprajacent vertebra, we have observed that in the 8th thoracic vertebra (T8) the left transverse process is taphonomically distorted and located too dorsally. Thus, we have slightly displaced the eight left rib (8L) to compensate this.

Ninth rib

The original ninth rib from the right side (9R) was used in the 3D thorax reconstruction. Due to the taphonomical distortion of the 9L, the mirror imaged of the 9R was used in the 3D thorax reconstruction.

Tenth rib

The original tenth rib from the right side (10R) was used in the 3D thorax reconstruction. It was only slightly reconstructed: a bone chip that was missing in the posterior angle was virtually filled. Due to the taphonomical distortion of the 10L, the mirror imaged of the slightly reconstructed 10R was used in the 3D thorax reconstruction.

Eleventh rib

We have detected a reconstruction error in the eleventh rib from the right side (11R). The morphology of the head and neck of 11R does not correspond to the morphology expected for this anatomical region. In fact, the neck is relatively long and cranio-caudally narrower than what one can expect from an 11th rib. The comparison with 11L and a close look at 11R results in the realization that the head and neck are a refitted fragment, and the morphology of the added fragment is consistent with that of a rib located more cranially. Thus, 11R suffers from an error in

the original reconstruction. In this study, as 11L preserves part of the head (which articulates with the eleventh thoracic vertebra), and thus we have mirror-imaged 11L to use it on both sides.

This error in the reconstruction was not perceived in previous studies[15,19]. This explains the differences of 11 mm of the head-ventral arc between 11R (231.0 mm) and 11L (220.0 mm) (Gómez-Olivencia et al.'s[15] Table 3). 11L preserves the correct measurement. The extra fragment (i.e., the head and the neck) should be removed in any analysis regarding 11R (Supplementary Fig. 19).

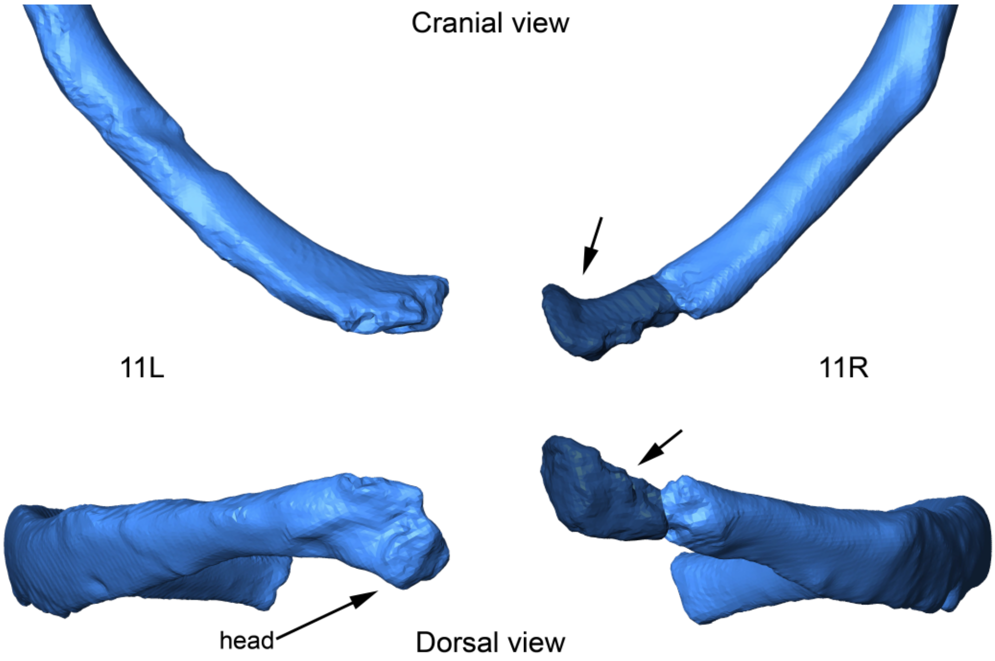

Supplementary Fig. 19. Cranial (top) and dorsal (posterior) views of the eleventh ribs from the left (L) and right (R) sides. The darker part in 11R (arrows) correspond to a fragment representing the head and part of the neck of a more cranially located rib which has been incorrectly refitted to 11R. This extra fragment should be removed in any analysis regarding this rib.

Twelfth rib

We have detected a reconstruction error in the twelfth rib from the right side (12R). When compared to its antimere (i.e., with 12L), this rib is longer. 12R is the result of the refitting of two fragments, the first of which represents the larger part of the rib and smaller fragment that represents a portion of the shaft. A comparison with 12L led us to the realization that the latter fragment does not follow the normal anatomy of the rib. The extra fragment should be removed in any analysis regarding this rib (Supplementary Fig. 20). In this study, as 12L is complete and thus we have mirror-imaged it to use it on both sides.

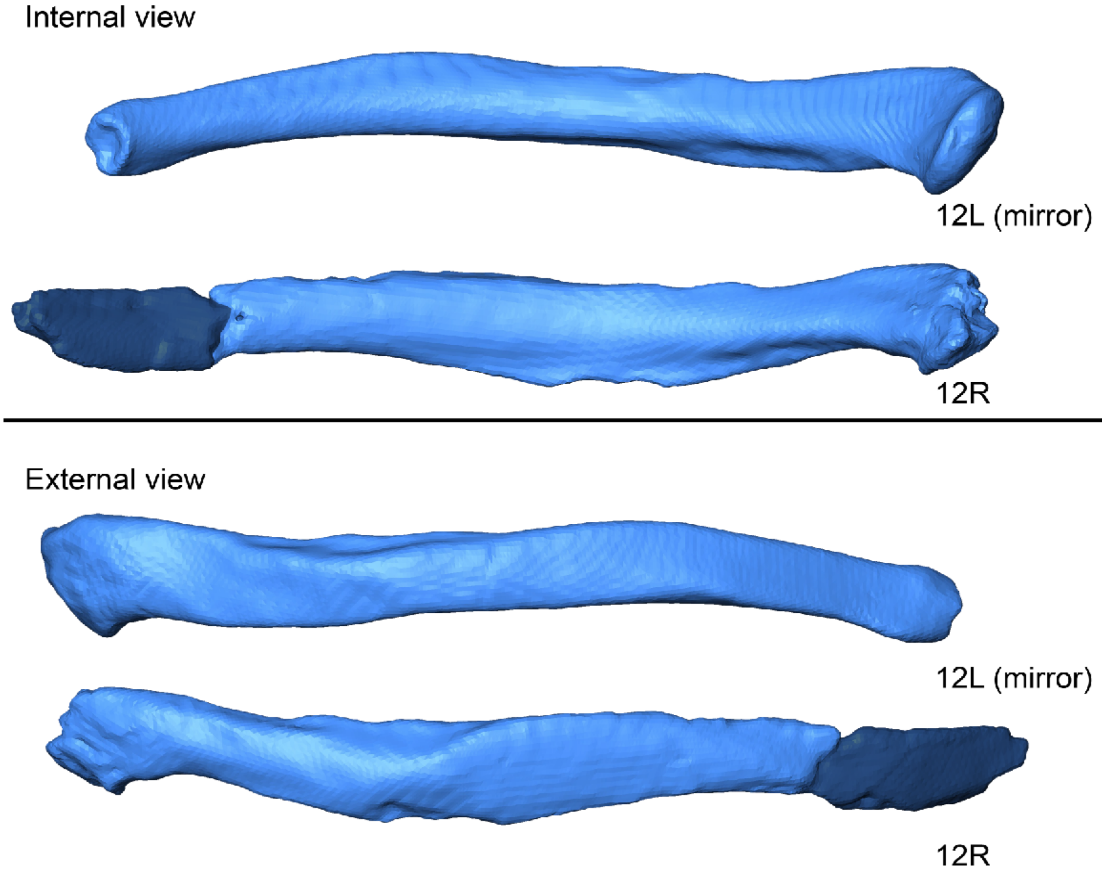

Supplementary Fig. 20. Internal (i.e., looking from within the thorax; top) and external (bottom) views of the twelfth ribs from the left (L) and right (R) sides. The darker part in 12R (arrows) correspond to a fragment representing a shaft fragment which does not properly refit to this rib and which makes it anomalously long. This extra fragment should be removed in any analysis regarding this rib.

SUPPLEMENTARY REFERENCES

1. Arensburg, B. *et al.* Une sépulture néandertalienne dans la grotte de Kébara (Israël). *C.R. Acad. Sci. Paris* **300**, 227–230 (1985).
2. Bastir, M. *et al.* Three-dimensional morphometrics of thoracic vertebrae in Neandertals and the fossil evidence from El Sidrón (Asturias, Northern Spain). *J. Hum. Evol.* **108**, 47–61 (2017a).
3. Been, E., Gómez-Olivencia, A., Kramer, P. A. & Barash, A. in *Human Paleontology and Prehistory: Contributions in Honor of Yoel Rak.* (Springer, 2017a).
4. Duday, H. & Arensburg, B. in *Le squelette moustérien de Kébara 2* (eds O. Bar-Yosef & B. Vandermeersch) 180–193 (Éditions du CNRS, 1991).
5. García-Martínez, D. *et al.* Reevaluation of "endocostal ossifications" on the Kebara 2 Neanderthal ribs. *J. Hum. Evol.* (accepted).
6. Gómez-Olivencia, A., Been, E., Arsuaga, J. L. & Stock, J. T. The Neandertal vertebral column 1: The cervical spine. *J. Hum. Evol.* **64**, 608–630 (2013b).
7. Trinkaus, E., Churchill, S. E. & Ruff, C. B. Postcranial robusticity in *Homo*. II: humeral bilateral asymmetry and bone plasticity. *Am. J. Phys. Anthropol.* **93**, 1–34 (1994).
8. Franciscus, R. G. & Churchill, S. E. The costal skeleton of Shanidar 3 and a reappraisal of Neandertal thoracic morphology. *J. Hum. Evol.* **42**, 303–356 (2002).
9. Arensburg, B. in *Le squelette moustérien de Kébara 2* (eds O. Bar-Yosef & B. Vandermeersch) 113–147 (Éditions du CNRS, 1991).
10. Peleg, S. *et al.* Orientation of the human sacrum: Anthropological perspectives and methodological approaches. *Am. J. Phys. Anthropol.* **133**, 967-977 (2007).
11. Been, E., Pessah, H., Peleg, S. & Kramer, P. A. Sacral orientation in hominin evolution. *Advances in Anthropology* **3**, 133-141 (2013).

12. Been, E., Gómez-Olivencia, A. & Kramer, P. A. Brief Communication: Lumbar lordosis in extinct hominins: Implications of the pelvic incidence. *Am. J. Phys. Anthropol.* **154**, 307–314 (2014).

13. Goh, S., Price, R. I., Leedman, P. J. & Singer, K. P. The relative influence of vertebral body and intervertebral disc shape on thoracic kyphosis. *Clinical Biomechanics* **14**, 439-448 (1999).

14. Rak, Y. & Arensburg, B. Kebara 2 Neanderthal pelvis: First look at a complete inlet. *Am. J. Phys. Anthropol.* **73**, 227–231 (1987).

15. Gómez-Olivencia, A., Eaves-Johnson, K. L., Franciscus, R. G., Carretero, J. M. & Arsuaga, J. L. Kebara 2: new insights regarding the most complete Neandertal thorax. *J. Hum. Evol.* 57, 75–90 (2009).

16. Gómez-Olivencia, A. *et al.* The costal skeleton of *Homo antecessor*: preliminary results. *J. Hum. Evol.* **59**, 620–640 (2010).

17. Gómez-Olivencia, A., Holliday, T., Madelaine, S., Couture-Veschambre, C. & Maureille, B. The costal skeleton of the Regourdou 1 Neandertal. *J. Hum. Evol.*, doi.org/10.1016/j.jhevol.2017.12.005 (in press).

18. Bastir, M. *et al.* The relevance of the first ribs of the El Sidrón site (Asturias, Spain) for the understanding of the Neandertal thorax. *J. Hum. Evol.* **80**, 64–73 (2015a).

19. García-Martínez, D. *et al.* On the chest size of Kebara 2. *J. Hum. Evol.* **70**, 69–72 (2014).
